# Infant urinary metabolomic profile in a fatal acute methadone intoxication

**DOI:** 10.1007/s00414-021-02772-z

**Published:** 2022-01-11

**Authors:** Alberto Chighine, Michele Porcu, Giulio Ferino, Nicola Lenigno, Claudia Trignano, Ernesto d’Aloja, Emanuela Locci

**Affiliations:** 1 Department of Medical Sciences and Public Health, Section of Legal Medicine, University of Cagliari, Cittadella Universitaria Di Monserrato, 09042 Monserrato, Cagliari, Italy; 2 Department of Medical Sciences and Public Health, Section of Radiology, University of Cagliari, Cagliari, Italy; 3 Department of Biomedical Sciences, University of Sassari, Sassari, Italy

**Keywords:** Asphyxial deaths, Metabolomics, ^1^H NMR, Forensics, Methadone intoxication, Urine

## Abstract

A case report suspicious for a Sudden Infant Death Syndrome is here described. Pathological findings were consistent with an acute respiratory failure while toxicological analysis revealed an elevated blood methadone concentration. Death was then ascribed to an acute methadone intoxication. In addition to the routinary approach, the urinary sample collected at autopsy was investigated with a ^1^H NMR metabolomic approach and the identified metabolomic profile was challenged with the urinary metabolomic profiles previously obtained from 10 newborns who experienced perinatal asphyxia and 16 healthy control newborns. Intriguingly, the urinary profile of the methadone intoxicated infant was very similar to those belonging to the perinatal asphyxia newborns, especially to those belonging to the newborns characterised by the worst outcome. The results offer several hints on a shared metabolic derangement between different mechanisms of asphyxia/hypoxia. To the best of the authors’ knowledge, this is the first report of the use of a metabolomic approach in a pathological case, in which metabolomics offers useful additional information regarding the mechanism and the cause of death.

## Introduction

Whenever a forensic pathologist must deal with an infant death, every case without clear and macroscopic evidence of a pathological condition or a violent death should be investigated in the suspicion of Sudden Infant Death Syndrome (SIDS). According to the San Diego definition, the SIDS diagnosis relies on the exclusion of any other possible cause of death, including toxicological investigations [1].

Methadone fatal and non-fatal intoxications were described in infants, often breastfed, in the population under 1 year of age, addressing several pathological, toxicological and pharmacological aspects of acute and chronic methadone intoxication [2].

In this work, we present a case of a 49-day-old male infant whose death was ascribed to acute methadone intoxication (MI). Pathological and toxicological data were collected. Post-mortem computed tomography (CT) was also performed. A ^1^H NMR metabolomics approach was performed to investigate a post-mortem urinary sample. The identified metabolomic profile was then compared to a peculiar urinary profile previously studied in a cohort of newborns who experienced perinatal asphyxia (PA) and healthy controls [3]. Important insights on the mechanism of MI death were obtained through this approach.

To the best of our knowledge, this is the first report in which metabolomics is used as a complementary tool to investigate the mechanism of death in a real-world scenario.

## Materials and methods

The medico legal investigation included a preliminary full-body post-mortem CT examination, external body examination and autopsy, during which biological samples were collected for laboratory analyses.

### Toxicological analyses

Toxicological analyses, aimed at identifying the presence of drugs, poisons and metabolites, were conducted on two biological matrices, namely cardiac blood and peritoneal fluid by gas chromatography-tandem mass spectrometry (GC–MS/MS). Sample analysis was performed on an Agilent Technologies 7890 gas chromatograph combined with an Agilent 7000D triple-quadrupole mass spectrometer (Agilent Technologies, USA). Blood ethanol concentration was measured by Head-Space GC coupled with MS (HS-GC/MS) employing the CTC Combi Pal Head-Space autosampler coupled with a Varian 431 GC and a Varian 220-MS as detector (Agilent Technologies, USA).

A salt-assisted liquid–liquid extraction method was used for samples preparation prior to GC–MS/MS analysis. General unknown analysis showed the presence of methadone both in blood and in peritoneal fluid. Methadone was exactly quantified in blood by using methadone-*d*_*3*_ as internal standard and referring to a calibration curve.

### Post-mortem imaging

A volumetric non-contrast whole-body computed tomography (CT) was acquired prior the autopsy. The scan was acquired on an 80-rows Toshiba Aquilion prime (Canon Medical Systems Corporation, Otawa, Japan), adopting the following technical parameter: acquisition plane: axial; kVp = 100; tube current: 200 mAs; matrix: 512 × 512; slice thickness: 1 mm; spiral pitch: 0.656.

### Metabolomics analyses

MI urinary metabolomic profile was compared with a previously analysed dataset of urinary samples from 10 full-term newborns who experienced PA collected at birth and at different time points after birth, and urinary samples collected at birth from 16 healthy control newborns as previously described [3]. The same protocol was here applied to the post-mortem urine sample collected from the MI infant during the autopsy. Soon after collection, the sample was immediately frozen and kept at –80 °C until NMR analysis. Before NMR, the sample was thawed and centrifuged at 15,750 g for 10 min to remove any solid debris. The supernatant was mixed with a 0.1% w/w aqueous solution of sodium azide (NaN_3_, Sigma-Aldrich, Milan, Italy) to avoid bacterial growth and a 1.5 M phosphate buffer solution (pH = 7.4) in D_2_O (99.9%, Cambridge Isotope Laboratories Inc., Andover, MA, USA) containing the internal standard sodium 3-(trimethylsilyl)propionate-2,2,3,3,-*d4* (TSP, 98 atom % D, Sigma-Aldrich, Milan) at a 0.58 mM final concentration was added. The obtained solution was then transferred into a 5-mm NMR tube.

^1^H NMR was performed on a Varian UNITY INOVA 500 spectrometer (Agilent Technologies, CA, USA) operating at 499.839 MHz for proton. The spectra were acquired using a standard 1D-NOESY pulse sequence for presaturation of the water resonance, with a mixing time of 1 ms and a recycle time of 6 s. Spectra were recorded at 300 K with a spectral width of 6000 Hz, a 90° pulse and 128 scans. Before Fourier transformation, the free induction decays (FID) were weighted by an exponential function equivalent to a line broadening of 0.5 Hz and zero-filled to 64 K All spectra were phased and baseline corrected using MestReNova software (Version 14.1.2, Mestrelab Research S.L.). Chemical shifts were referred to the TSP single resonance at 0.00 ppm.

The spectral region 1.16–9.00 ppm was reduced into consecutive bins of 0.02 ppm width using MestReNova. To minimise the effects of variable concentration among different samples, for each spectrum, the integrated areas within each bin were normalised to a constant sum of 100. The region containing the residual water signal was excluded before integration. The final matrix where the MI spectral data were merged with the previously analysed dataset of PA and healthy newborns was imported into the SIMCA software (Version 14.0, Umetrics, Umea, Sweden) for multivariate analysis. Mean centering and Pareto scaling were applied prior to perform data analysis. Exploratory principal component analysis (PCA) was performed to identify peculiar clusters, anomalies or trends in the samples based on the similarities of their metabolic profiles. The number of components for the models was optimised using sevenfold cross validation. Agglomerative hierarchical cluster analysis (HCA) was also applied. Distance between clusters in the multivariate space was measured according to Ward’s linkage, and the results were plotted as a tree plot, where the vertical axis indicates the increase of variance.

## Results

### Pathological and post-mortem imaging findings

External examination was negative for macroscopic malformations and any kind of lesions, with particular respect to external petechiae. Livores were localised exclusively on the back side. The recorded anthropometrical parameters (weight, length and head circumference) were suggestive of a postnatal growth retardation [4], as shown in Table 1.

**Table 1:** Anthropometrical parameters recorded at birth and at autopsy (49° day). Percentile values according to WHO growth charts are reported in square brackets

	Weight (g)	Length (cm)	HC^a^ (cm)
At birth	2980 [21°]	48 [21°]	32 [2.5°]
At autopsy	3446 [15.3°]	53 [1.8°]	35 [1.6°]

^**a**^Head circumference

The autopsy revealed *situs solitus* and no macroscopical visceral malformations with the sole exception of a left kidney hypoplasia. Organ weights are reported in Table 2.

**Table 2:** Organ weights recorded at autopsy

	Weight (g)
Brain	560
Thymus	22
Heart	21
Right lung	46.5
Left lung	41
Liver	119
Spleen	13
Right kidney	23.5
Left kidney	3

Several petechial haemorrhages were found on the thymus, on the serous surface of both pleurae and on the epicardium. A dense white foam was found in the larynx, trachea and bronchi. On the section, the pulmonary parenchyma appeared homogeneous, intensely congested and, when pressed, released a combination of a white foam and blood.

**Fig. 1:**
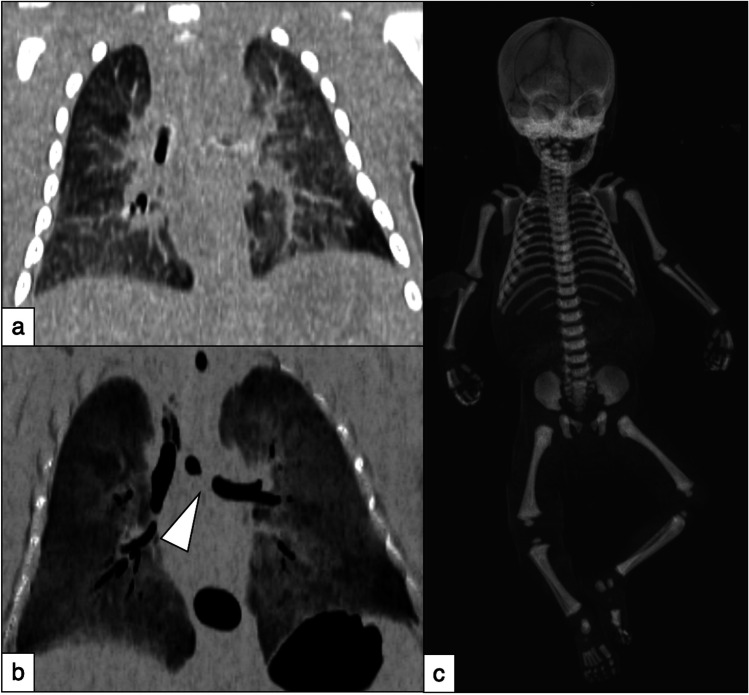
Post-mortem non-contrast CT. (a) Reconstructions at maximum intensity projection (4 mm thickness – coronal view) show diffuse inhomogeneity on both lungs with a diffuse ground glass opacifications and tiny air space consolidations as for alveolar oedema. (b) Chest details: reconstructions at minimum intensity projection (4 mm thickness – coronal view) confirm a diffuse ground glass opacifications on both lungs. Left main bronchus is partially occluded by mucoid secretions (white arrowhead). (c) Volume rendering reconstructions: no signs of traumatic and/or pathological fractures

CT chest scans revealed a diffuse ground glass opacifications and tiny air space consolidations as for alveolar oedema; trachea and left main bronchus were partially filled with secretions (Fig. 1a and 1b). Scans were negative for signs of traumatic fractures in the whole body (i.e. greenstick fractures, Fig. 1c), intracranial and/or intrabdominal abnormalities. A bilateral mammary hypertrophy was also detected.

### Histopathological findings

Histopathological analyses were performed on the major organs, and results were consistent with a severe acute respiratory insufficiency characterised by an alternation of areas of parenchymal consolidation with broken septa and partially collapsed alveoli occupied by a plasma-like material. No alterations were found in the remaining organs.

### Toxicological findings

Toxicological analysis performed via GC–MS/MS revealed the presence of methadone in the analysed biological matrices, namely heart blood and peritoneal fluid. An exact blood concentration of 570 ng/mL was then quantified.

### Metabolomics results

**Fig. 2:**
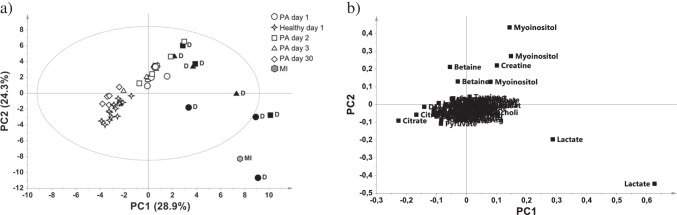
PCA model of all the urinary samples. (a) Score plot of PC1 vs PC2 of the samples collected from the MI infant at autopsy (grey diamond), PA newborns at birth (PA day 1, open circles), at 48 h (PA day 2, open squares), at 72 h (PA day 3, open triangles), at 30 days (PA day 30, open diamonds) and from healthy control newborns at birth (open stars). (b) PCA loading plot of the variables (metabolites) responsible for the separation of the samples in the corresponding score plot. Most of metabolites clustered together, representing the ‘normal’ metabolome composition. Metabolites on the verges are the ones driving the sample separation. The first three PCs explained the 63.3% of the total variance. In the score plot, non-surviving PA babies are labelled with the letter D and the corresponding symbols are plain black

As previously reported, 3 out of the 10 asphyxiated infants died in the first week of life due to severe PA complications and showed, already at birth, a peculiar urinary metabolome [3]. An exploratory PCA analysis was applied to all the collected samples to investigate possible similarities or differences among the samples based on their metabolomic profiles (Fig. 2). The PCA score plot (Fig. 2a) shows that the MI urine sample lays in the same multivariate space of the PA samples, and, interestingly, clusters with the subgroup of urines collected at birth from the asphyxiated newborns who eventually died, indicating a very similar urinary metabolome composition and, therefore, similar metabolomic profiles between MI and the non-surviving PA samples.

The great majority of variables representing metabolites clustered together in the loading plot (Fig. 2b) since they represent a backbone of the human metabolome in similar individuals. At the same time, particular circumstances may be responsible of slight but significative differences of relatively few metabolites, which appear more discernible in the graphical representation.

In the presented results, the separation was mainly driven by lactate, which increased in both MI infant and the non-surviving PA newborns’ urines as shown by the PCA loading plot.

**Fig. 3:**
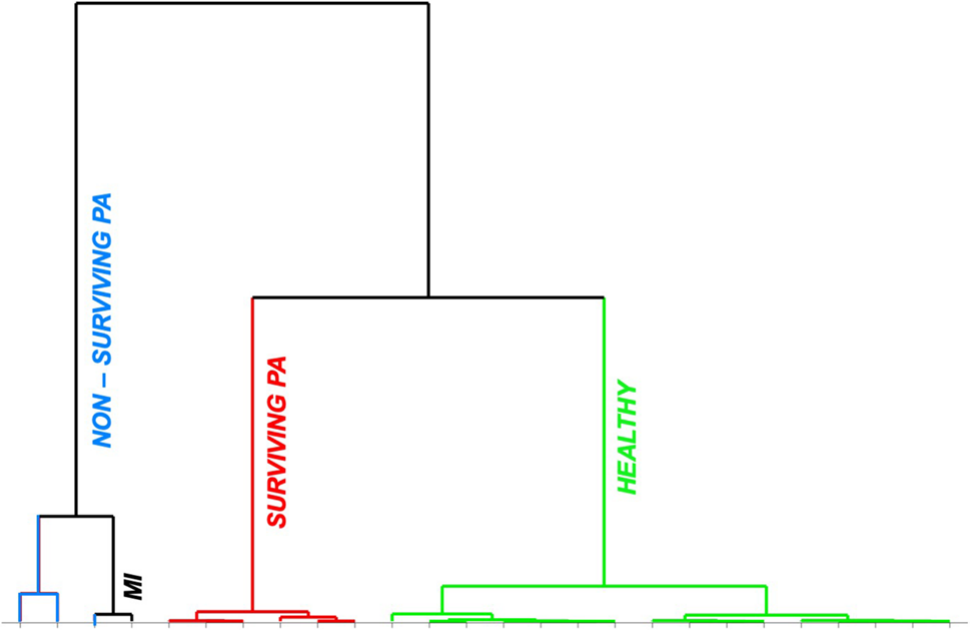
Hierarchical cluster analysis, sorted by Ward distance, tree plot of sample distances in the space spanned by the first two PCs. Samples collected from the MI infant at autopsy (black), PA newborns at birth who will eventually die (blue), PA newborns at birth (red) and healthy control newborns at birth (green). MI lies in the same node of PA newborns with the worst outcome, being far away from both PA with a good outcome and healthy controls

The PCA was run again excluding all the PA urines collected after day 1 focusing only on the PA samples collected at birth. The results are shown as an agglomerative HCA sorted by Ward distance in the space spanned by the first two components (Fig. 3). The obtained tree plot of sample distances indicates a first node where the non-surviving PA newborns together with MI samples are clearly separated from the surviving PA and healthy newborns’ samples. A second node further indicates the separation between healthy and asphyctic newborns with good outcome. Three group of samples are thus identifiable according to the similarities in their metabolomic profiles: the healthy control group, the surviving PA newborns and the non-surviving PA newborns together with the MI infant. This representation reinforces the observation that MI urinary profile is very similar to the severe asphyxial ones, clustering in the same node of PA newborns with the worst outcome.

## Discussion

Methadone intoxication in paediatric population is a matter of concern for several reasons. Firstly, the dose–effect relationship is stronger than in the adult population. Secondly, the interindividual variability highlighted in the adult methadone liver metabolism has not been well established in the paediatric population [5, 6]. Lastly, methadone has been associated with a prolonged QT interval, increasing the risk of sudden death [7].

While reports of fatal intoxication in the paediatric population are relatively common [8], to the best of our knowledge, only few cases regarding infants under 1 year have been described in the literature up to this date [2, 9].

Death circumstances and age raised the suspicion of a SIDS. The diagnosis of this complex syndrome relies on an exclusion criterion: SIDS as a cause of death diagnosis might be determined only when all other causes have been excluded [1, 10].

In this case, pathological findings and post-mortem imaging [11, 12] suggested a mechanism of death (acute respiratory failure) coherent with a SIDS diagnosis, as it often features pulmonary findings. Petechial haemorrhages found sub-pleurally, sub-epicardially and in the thymus were also consistent with a SIDS [13].

On the contrary, the toxicological investigation detected a positivity for the drug methadone, with a blood concentration of 570 ng/mL, allowing to rule out the diagnosis of SIDS. Such concentration appears to be in the range described as toxic/fatal [14, 15] and it is consistent with a mechanism of death of acute respiratory failure through the respiratory drive inhibition. What is more, being therapeutic and toxic level mostly referred to adults, effects are reasonably magnified in the paediatric population. Nevertheless, the determined concentration lies in the range detected in fatal cases in infants under 1 year (ranging from 69 to 700 ng/mL) [2].

Omics sciences, following their large application in clinical medicine [16] are increasingly used in the forensic setting [17–19]. Recently, our research group reviewed previous, current and future potential applications of NMR metabolomics in the forensic field [20], while several works deal with metabolomics in perinatal asphyxia, in human and animal models [21–23].

As pathological and toxicological findings were consistent with an acute respiratory insufficiency, the identified urine metabolomic profile was compared with previous metabolomic data of newborns who experienced PA [3]. Such comparison relies either on the similar age or on the hypothesis that different causes of asphyxia/hypoxia may share a common metabolic derangement.

Metabolomic profiles of individuals experiencing an acute hypoxic event were clearly distinguishable from the healthy ones. Major metabolomic modifications were the increase in urinary lactate and the decrease in myo-inositol, betaine and taurine. Among the hypoxic profiles, a further stratification was evidenced between survivors and those who eventually died, being the MI infant profile clustered with the latter. This correlation seems to be mainly driven by a single metabolite, namely lactate whose increase may be explained with a substantial switch to the anaerobic glycolysis, in the lack of oxygen. Data indicate a shared derangement in the metabolome between different mechanisms of asphyxia/hypoxia.

To the best of our knowledge, this is the first report of a fatal methadone intoxication under 1 year of age in which, in addition to classical findings, a urinary metabolomic investigation together with a post-mortem full-body CT scan was provided. In particular, the ^1^H NMR approach allowed not only to strengthen the causal link between methadone blood levels and the acute respiratory failure, but also provided evidence of the asphyxial perturbation on metabolome, independently on its origin. The latter reinforces strong asphyxial evidence on animal models [24, 25] and hints its translation on the human model on several biological matrices, towards the implementation of metabolomics as an additional, powerful, diagnostic tool in the forensic scenario.

The results are of great forensic relevance. Data suggests a shared hypoxic signature, highlighting the potential of metabolomics as an additional tool to investigate different causes and mechanisms of death.

This study has several limitations. Post-mortem CT scan was conducted without contrast medium. Metabolomic was performed on urine, needing a reference dataset of a comparable population, as urine is the biofluid of choice in the perinatal clinical settings, due to ethical reasons. More biofluids (i.e. serum and vitreous humour) should be investigated on these purposes. Although metabolic perturbation might be shared, the comparison group chosen underwent a different cause of hypoxia. The effect of post-mortem interval (PMI) on the metabolome was not taken into account [26]. Whereas part of the modifications in the MI may be driven by PMI, MI and PA metabolomic similarities are strengthened by PA modifications occurring in living individuals. Toxicological analysis was performed on cardiac blood as just a residual amount was found peripherally. Despite the aforementioned limitations, we believe these results may pave the way for the implementation of novel scientific disciplines in the forensic field.

## Data Availability

All raw data are available upon request.

## References

[1] Bajanowski T, Brinkmann B, Vennemann M (2006). The San Diego definition of SIDS: practical application and comparison with the GESID classification. Int J Legal Med.

[2] Paul ABM, Simms L, Mahesan AM (2017). The toxicology of methadone-related death in infants under 1 year: three case series and review of the literature. J Forensic Sci.

[3] Locci E, Noto A, Puddu M, Pomero G, Demontis R, Dalmazzo C, Delogu A, Fanos V, d’Aloja E, Gancia P (2018). A longitudinal 1H-NMR metabolomics analysis of urine from newborns with hypoxic-ischemic encephalopathy undergoing hypothermia therapy. Clinical and medical legal insights. PLoS One.

[4] WHO Multicentre Growth Reference Study Group (2006). WHO Child Growth Standards based on length/height, weight and age. Acta Paediatr Suppl.

[5] Wang JS, DeVane CL (2003). Involvement of CYP3A4, CYP2C8, and CYP2D6 in the metabolism of (R)- and (S)-methadone in vitro. Drug Metab Dispos.

[6] Giorgetti A, Pascali J, Montisci M, Amico I, Bonvicini B, Fais P, Viero A, Giorgetti R, Cecchetto G, Viel G (2021). The role of risk or contributory death factors in methadone-related fatalities: a review and pooled analysis. Metabolites.

[7] Eap CB, Crettol S, Rougier JS, Schläpfer J, Sintra Grilo L, Déglon JJ, Besson J, Croquette-Krokar M, Carrupt PA, Abriel H (2007). Stereoselective block of hERG channel by (S)-methadone and QT interval prolongation in CYP2B6 slow metabolizers. Clin Pharmacol Ther.

[8] Bonsignore A, Groppi A, Ventura F, De Stefano F (2016). Palmiere C (2016) Fatal methadone intoxication in an infant listed as a homicide. Int J Legal Med.

[9] Madadi P, Kelly LE, Ross CJ, Kepron C, Edwards JN, Koren G (2016). Forensic investigation of methadone concentrations in deceased breastfed infants. J Forensic Sci.

[10] Bajanowski T, Vennemann M, Bohnert M, Rauch E, Brinkmann B, Mitchell EA, GeSID Group (2005). Unnatural causes of sudden unexpected deaths initially thought to be sudden infant death syndrome. Int J Legal Med.

[11] Tsuchiya N, Griffin L, Yabuuchi H, Kawanami S, Shinzato J, Murayama S (2020). Imaging findings of pulmonary edema: Part 1. Cardiogenic pulmonary edema and acute respiratory distress syndrome. Acta Radiol.

[12] Tsuchiya N, Griffin L, Yamashiro T, Gibo S, Okane T, Yasutani T, Murayama S (2020). Imaging findings of pulmonary edema: Part 2. Infrequent or unusual pulmonary edema with definitive imaging findings. Acta Radiol.

[13] Fracasso T, Vennemann M, Klöcker M, Bajanowski T, Brinkmann B, Pfeiffer H, Bach P, Bockholdt B, Bohnert M, Cremer U, Deml U, Freislederer A, Heide S, Huckenbeck W, Jachau K, Kaatsch HJ, Klein A, Kleemann WJ, Larsch KP, Fieguth A, Leukel HW, Mützel E, Rublack F, Sperhake J, Zimmer G, Zweihoff R, GeSID Group (2011). Petechial bleedings in sudden infant death. Int J Legal Med.

[14] Musshoff F, Padosch S, Steinborn S, Madea B (2004). Fatal blood and tissue concentrations of more than 200 drugs. Forensic Sci Int.

[15] Schulz M, Schmoldt A, Andresen-Streichert H, Iwersen-Bergmann S (2020). Revisited: Therapeutic and toxic blood concentrations of more than 1100 drugs and other xenobiotics. Crit Care.

[16] Nicholson JK, Holmes E, Kinross JM, Darzi AW, Takats Z, Lindon JC (2012). Metabolic phenotyping in clinical and surgical environments. Nature.

[17] Merkley ED, Wunschel DS, Wahl KL, Jarman KH (2019). Applications and challenges of forensic proteomics. Forensic Sci Int.

[18] Ferreira PG, Muñoz-Aguirre M, Reverter F, Sá Godinho CP, Sousa A, Amadoz A, Sodaei R, Hidalgo MR, Pervouchine D, Carbonell-Caballero J, Nurtdinov R, Breschi A, Amador R, Oliveira P, Çubuk C, Curado J, Aguet F, Oliveira C, Dopazo J, Sammeth M, Ardlie KG, Guigó R (2018). The effects of death and post-mortem cold ischemia on human tissue transcriptomes. Nat Commun.

[19] Lyu L, Sonik N, Bhattacharya S (2021). An overview of lipidomics utilizing cadaver derived biological samples. Expert Rev Proteomics.

[20] Locci E, Bazzano G, Chighine A, Locco F, Ferraro E, Demontis R, d’Aloja E (2020). Forensic NMR metabolomics: one more arrow in the quiver. Metabolomics.

[21] Locci E, Bazzano G, Demontis R, Chighine A, Fanos V, d’Aloja E (2020). Exploring perinatal asphyxia by metabolomics. Metabolites.

[22] Denihan NM, Boylan GB, Murray DM (2015). Metabolomic profiling in perinatal asphyxia: a promising new field. Biomed Res Int.

[23] Sachse D, Solevåg AL, Berg JP, Nakstad B (2016). The role of plasma and urine metabolomics in identifying new biomarkers in severe newborn asphyxia: a study of asphyxiated newborn pigs following cardiopulmonary resuscitation. PLoS ONE.

[24] Varvarousis D, Xanthos T, Ferino G, Noto A, Iacovidou N, Mura M, Scano P, Chalkias A, Papalois A, De-Giorgio F, Baldi A, Mura P, Staikou C, Stocchero M, Finco G, d’Aloja E, Locci E (2017). Metabolomics profiling reveals different patterns in an animal model of asphyxial and dysrhythmic cardiac arrest. Sci Rep.

[25] Locci E, Chighine A, Noto A, Ferino G, Baldi A, Varvarousis D, Xanthos T, De-Giorgio F, Stocchero M, d’Aloja E (2021). Metabolomics improves the histopathological diagnosis of asphyxial deaths: an animal proof-of-concept model. Sci Rep.

[26] Chighine A, Locci E, Nioi M, d’Aloja E (2021). Looking for post-mortem metabolomic standardization: waiting for godot-the importance of post-mortem interval in forensic metabolomics. Chem Res Toxicol.

